# 磁增强管内固相微萃取的材料制备及应用进展

**DOI:** 10.3724/SP.J.1123.2024.05030

**Published:** 2025-06-08

**Authors:** Yana LUO, Jia CHEN, Yuyu HU, Shijie GAO, Yanli WANG, Yanming LIU, Juanjuan FENG, Min SUN

**Affiliations:** 1.济南大学化学化工学院，山东 济南 250022; 1. School of Chemistry and Chemical Engineering，University of Jinan，Jinan 250022，China; 2.山东省食品药品检验研究院，山东省食品药品安全检测工程技术中心，山东 济南 250101; 2. Shandong Institute for Food and Drug Control，Shandong Research Center of Engineering and Technology for Safety Inspection of Food and Drug，Jinan 250101，China

**Keywords:** 磁增强管内固相微萃取, 样品前处理, 环境分析, 食品分析, 生物医药分析, 综述, magnetism-enhanced in-tube solid-phase microextraction （ME-IT-SPME）, sample preparation, environmental analysis, food analysis, biomedicine analysis, review

## Abstract

样品进行分析检测时因基质成分复杂、分析物含量较低，直接用仪器进行准确、灵敏分析存在困难，因此在样品进行色谱分析检测前必须选择合适的样品前处理方法以实现目标物的选择性富集。在众多的样品前处理方法中，管内固相微萃取（IT-SPME）具有微型化、环境友好等优点，是一种有发展前景的样品前处理技术。萃取效率不理想、萃取速率慢等不足限制了IT-SPME的快速发展。为了进一步提高萃取的选择性和萃取效率，改善IT-SPME存在的不足，减少样品损失和污染，外加磁场力作用被引入IT-SPME，发展出磁增强管内固相微萃取（ME-IT-SPME）新技术。纳米材料、整体材料、磁性杂化材料等新型萃取材料主要通过亲/疏水作用、氢键、*π*-*π*作用、极性相互作用、配位作用等相互作用选择性吸附和高效富集多种类型的分析物，如有机农药、重金属离子、除草剂、雌激素、防腐剂、药物分子等。ME-IT-SPME在吸附和洗脱阶段对微萃取管（开管柱型、颗粒填充型、整体柱型）施加不同强度和方向的磁场，显著地提高了萃取效率，并联用色谱法对目标物进行高灵敏分析，获得了准确的分析结果，有效节约了实验成本，实现了高通量和快速准确的自动化分析。本文介绍了自2012年ME-IT-SPME技术诞生以来，ME-IT-SPME的材料制备，综述了ME-IT-SPME技术在环境分析、食品检测和生物医药领域的应用进展，并对该技术的未来发展进行了展望。

## Abstract (en)

Selecting a suitable sample preparation method is a significant step prior to chromatographic separation and detection. Directly analyzing samples instrumentally is difficult owing to the complexity of the sample matrix and the trace concentration of analytes. Most sample preparation methods have disadvantages， including complicated operating procedures， the use of large amounts of organic solvent， and ease of analyte loss during multistep processes； consequently， they do not meet the high analytical sample detection requirements of modern industry. The development of simple， environmentally friendly， efficient， and rapid preparation methods is a continuing frontier research area in the analytical chemistry field. Among the many available sample preparation techniques， in-tube solid-phase microextraction （IT-SPME） is receiving extensive attention. IT-SPME enriches the target analytes by extracting them to the inner surface of the capillary tube， and has been applied to extract various analytes in the environmental and food fields. IT-SPME is advantageous because it consumes low amounts of organic solvent and capillaries are mechanical stable； consequently， IT-SPME is a promising sample preparation technique. Magnetic field has been introduced to the IT-SPME system to further improve extraction efficiency and selectivity， leading to the development of magnetism-enhanced in-tube solid-phase microextraction （ME-IT-SPME） as a new technology. ME-IT-SPME uses magnetic field to separate and enrich targets， with different magnetic-field strengths applied to the extraction column during adsorption and elution. Diamagnetic substances in a paramagnetic medium tend to concentrate in regions where the magnetic field is weak when an external magnetic field is applied. Target analytes are detected chromatographically following elution. Conditions are optimized and an analytical method is established and used to detect targets in actual samples， leading to improved extraction sensitivity and precision compared to those obtained using IT-SPME， including shorter analysis time and superior extraction efficiency. This paper reviews the applications of ME-IT-SPME technology in combination with various analytical instruments since its inception in 2012， and analyzes its analysis and detection advantages. Based on hydrophobic interactions， hydrogen bonding， *π*-*π* and polarity interactions， coordination， and other extraction mechanisms with analytes， ME-IT-SPME uses innovative functional extraction materials， including nanomaterials， monolithic materials， and magnetic hybrid materials， all of which have high surface areas and numerous adsorption sites. Capillary microextraction columns are prepared using open-tubular capillary， particle-filling capillary， or monolithic capillaries. Diverse analytes are detected when ME-IT-SPME is combined with chromatograph， including organic pesticide residues， heavy-metal ions， herbicides， preservatives， and drug molecules. ME-IT-SPME technology is widely used in the environmental-analysis， food-analysis， and biomedical fields. Future， ME-IT-SPME technological developments should include： （1） focus on the reusability and stability of the magnetic extraction material； （2） discovering new extraction materials that are highly enriching and selective in order to analyze a greater variety of targets； （3） further innovating ME-IT-SPME technology by combining it with other more-sensitive analytical methods and considering its use in other fields； （4） connecting different capillaries to simultaneously enrich a variety of analytes； （5） exploring how the higher magnetic field influences extraction efficiency by designing new magnetic-field-regulating devices with small thermal interference； （6） combining the technology with advanced portable analytical instruments to realize real-time target analysis in the field； （7） exploiting immuno-affinitive extraction tubes that can be used to highly efficiently and selectively extract biological macromolecular drugs.

由于样品基质复杂且分析物含量较低，在进行色谱分析检测前需要用合适的样品前处理方法实现分析物的选择性富集，从而获得准确的分析结果。大部分样品前处理方法需要使用大量有机溶剂，且多步过程易造成分析物损失，不能满足现代分析行业对样品检测的要求。因此，发展操作简便、绿色环保、高效快速的前处理方法一直是分析化学的研究热点。

近年来，固相微萃取（SPME）技术以简单、低成本等取代了传统的萃取方法。SPME基于分析物在样品基质和吸附剂之间的分配平衡，可产生定量或半定量结果^［1］^。SPME包括管内固相微萃取（IT-SPME）、纤维固相微萃取、薄膜固相微萃取、搅拌棒固相微萃取等，具有快速、灵敏度高等优点。此外，SPME实现了微型化，并能与各种分析仪器联用。这些优点使得SPME在众多的样品前处理方法中脱颖而出。SPME已被应用在环境分析、食品样品、生物化学等领域^［2］^。我们课题组^［3］^以离子液体（ILs）和正硅酸四乙酯（TEOS）为前驱体制备了聚离子液体杂化的二氧化硅气凝胶，将其涂覆在不锈钢丝表面，得到SPME纤维；结合气相色谱-氢火焰离子化检测器（GC-FID），建立了SPME-GC-FID富集检测实际水样中痕量多环芳烃（PAHs）的方法，获得了宽的线性范围和满意的加标回收率，且该萃取涂层具有重复性好等优点。Ali课题组^［4］^合成了含氧化钆纳米粒子的聚（3-羟基丁酸酯）-聚（甲基丙烯酸二甲基氨基乙酯）两亲性嵌段共聚物，用作SPME吸附剂。通过原子吸收光谱法测定小麦等食品中的总无机砷，得到了较高的富集倍数和良好的精密度。

IT-SPME不仅具有SPME的优点，还有利于与HPLC在线联用，使从样品制备到目标物的分离检测整个过程实现自动化，有效地减少样品的污染损失和提高分析结果的准确度，克服了SPME纤维易受损、萃取涂层易脱落和吸附容量低的缺点，实现了对目标物的富集^［5］^。IT-SPME可用于确定复杂基质中超痕量的目标分析物，是一种微型化、绿色环保的技术。IT-SPME现已被应用于环境样品中多种分析物的富集。我们课题组^［6］^在玄武岩纤维表面原位制备了一种与聚氨酯杂化的二氧化硅气凝胶，用于IT-SPME。将填充改性玄武岩纤维的聚醚醚酮管与HPLC-DAD在线联用，建立了环境样品中雌激素的IT-SPME-HPLC-DAD在线检测方法，获得了较低的检出限和较宽的线性范围。Ma等^［7］^通过化学氧化法在聚四氟乙烯管负载材料上制备了十二烷基苯磺酸掺杂聚吡咯的IT-SPME涂层，结合HPLC-MS/MS应用于环境水体中19种含氮农药的富集检测。结果表明，该新型萃取涂层显著提高了环境水中极性含氮农药的萃取效率。IT-SPME还可以应用于复杂的食品样品中痕量目标物的富集萃取。我们课题组^［8］^制备了氧化石墨烯功能化介孔二氧化硅，作为IT-SPME的涂层；与HPLC-DAD在线联用，建立了在线检测蜂蜜样品中痕量PAHs的灵敏方法，获得了令人满意的结果且该萃取管表现出良好的化学稳定性。基于该萃取材料的高效萃取性能，所建立的IT-SPME-HPLC-DAD在线分析方法灵敏度高，线性范围宽。近年来，开发能够增强分析物亲和力的独特吸附剂，并利用磁、电和热能的场效应增强萃取效率是分析化学的研究热点。磁力不需要大型的仪器仪表，并且磁力相互作用通常与离子强度和表面电荷无关，可以控制系统中流体运动。相较于一般的吸附剂，磁性萃取材料具有超顺磁性，在无外加磁场作用时几乎不表现磁性^［9］^。在外加磁场作用下，顺磁介质中抗磁性物质倾向于集中在磁场强度弱的区域，利用反磁力作用实现抗磁性物质的分离富集^［10］^。基于此原理，结合磁性萃取材料的优势，将外加磁场力引入IT-SPME中，发展出了磁增强管内固相微萃取（ME-IT-SPME）技术。ME-IT-SPME通过在吸附和洗脱阶段利用外加电源对萃取柱施加不同强度和方向的磁场，提高了萃取效率和萃取容量，洗脱后再通过色谱仪器检测目标物，减少了样品污染和损失，缩短了分析时间，有效节约了实验成本，获得了更加准确的分析结果。本文介绍了自2012年ME-IT-SPME技术诞生以来，ME-IT-SPME的材料制备，重点阐述了ME-IT-SPME技术在环境分析、食品检测和生物医药领域的应用进展，并对该技术的未来发展进行了展望。

## 1 磁性固相微萃取管的制备

ME-IT-SPME的吸附剂由毛细管内表面涂层或管内部填充萃取材料组成，通过对其进行预处理，保证了萃取柱床的稳定性。开管柱、颗粒填充柱、整体柱被用来制备ME-IT-SPME毛细管微萃取柱。萃取材料决定了ME-IT-SPME的萃取性能。基于磁性金属氧化物纳米颗粒的纳米材料、有机聚合物整体柱的整体材料、聚离子液体修饰掺杂磁性粒子整体柱的磁性杂化材料等新型萃取材料被用作ME-IT-SPME的吸附剂，以提高ME-IT-SPME的萃取性能。

### 1.1 开管柱型微萃取管

Moliner-Martínez等^［11］^通过溶胶-凝胶法合成了Fe_3_O_4_纳米粒子（nanoparticles，NPs）嵌入二氧化硅的杂化材料。将其沉积在75 μm的裸露熔融石英毛细管柱内，得到了10 μm厚的Fe_3_O_4_-SiO_2_萃取涂层。由于溶胶-凝胶法保证了Fe_3_O_4_ NPs的稳定性，二氧化硅的基质避免了纳米粒子相互团聚的现象。Fe_3_O_4_ NPs分散均匀且磁性良好。该涂层高度多孔的互穿网络结构为萃取提供了良好的传质效果。但是，聚合涂层的开管柱型固相微萃取管存在萃取容量有限的缺点，因此，发展了磁性吸附颗粒填充型固相微萃取管。

### 1.2 颗粒填充型微萃取管

Manbohi等^［12］^通过化学共沉淀法在N_2_气氛下将Fe^2+^、Fe^3+^、盐酸溶于纯水中制成混合液，再逐滴加入到NaOH溶液中，将得到的沉淀在磁场下从反应介质中分离，冲洗得到Fe_3_O_4_ NPs悬浮液。用注射器将NPs引入不锈钢毛细管中，在管周围放置强磁铁，使Fe_3_O_4_ NPs固定在管内。Fe_3_O_4_ NPs具有粗糙的结构和良好的形状，直径为75～85 nm。由于Fe_3_O_4_ NPs表面的单一性容易聚集，不利于吸附，采用表面活性剂十二烷基硫酸钠对其进行修饰以有效吸附分析物。填充了磁性纳米颗粒的固相微萃取管易与现有的自动取样器配合使用。该方法制备的磁性纳米材料尺寸粒径小，比表面积大，可以共价固定在毛细管内表面，从而为目标分子提供更多的吸附活性位点。然而，磁性吸附颗粒填充型固相微萃取管易被样品杂质堵塞，扩散传质速度慢且装填制备过程较繁琐困难。因此，分析工作者研发了制备简单且使用寿命长的整体柱型固相微萃取管。

### 1.3 整体柱型微萃取管

整体柱具有双孔结构、传质快、渗透性良好、萃取能力优良等优点。大孔保证了整体柱的渗透性，中孔增加了其表面积，可提供更多的活性吸附位点，能够实现高选择性的富集和高通量分析。Mei等^［13，14］^分别用4-乙烯基吡啶（VP）、4-乙烯基苯硼酸（VA）为功能单体（见图1），乙二醇二甲基丙烯酸酯（ED）为交联剂，并加入改性的磁性纳米粒子（MNPs）Fe_3_O_4_@SiO_2_@*γ*-MAPS，连续超声使纳米粒子分散均匀。将其填充到内表面被3-（三甲氧基甲硅基）甲基丙烯酸丙酯（*γ*-MAPS）修饰的熔融石英毛细管中，毛细管的末端密封并发生聚合反应，合成了两种嵌入Fe_3_O_4_ MNPs的毛细管整体柱（VPED-MCC/MNPs和MCEN）。相比VP，含有硼酸基团的VA作单体，可以利用吸附剂与目标物间的B*-*N键进行更好地萃取。在VPED-MCC/MNPs中，用*γ*-MAPS对熔融的石英毛细管预处理可以在内壁产生丙烯酸双键，丙烯酸双键可以参与聚合，使得聚合物整体柱床与毛细管内壁形成共价键合，并能够保证MNPs的稳定键合和均匀分布，提高了该整体柱的稳定性。在不损失萃取性能的情况下，VPED-MCC/MNPs使用了200次以上，表现出令人满意的使用寿命。在MCEN中，柱床呈现块体均匀的球状和多孔结构，Fe_3_O_4_ NPs均匀分散并嵌在其中，保证了MCEN良好的渗透性和机械稳定性。磁化曲线表明嵌入在MCEN中的Fe_3_O_4_ NPs具有超顺磁性，因此在外加磁场下可以很容易被磁化和退磁。

**图1:**
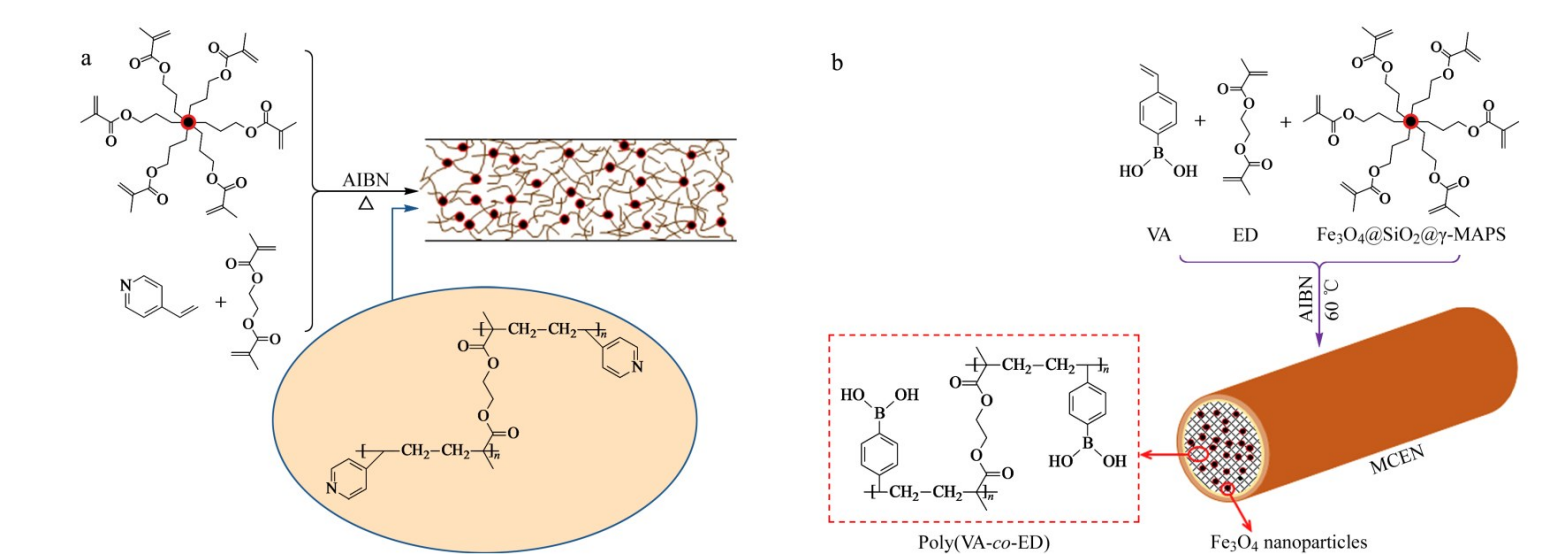
（a）VPED-MCC/MNPs的制备图^［13］^和（b）MCEN的聚合反应图^［14］^

适当的衍生化或改性处理可改变整体材料的表面性质，使其对目标物的萃取选择性能得到进一步改善。IL具有低蒸汽压、高热稳定性和可调的物理化学性质等特点，是对环境有害的传统有机溶剂的替代品，在样品制备领域得到了广泛关注。直接涂覆的方式易造成IL的流失，采用聚合接枝的方式，可以将其与其他材料形成稳定的复合物。Mei等^［15］^通过原位热引发聚合法合成了基于掺杂磁性纳米粒子的聚离子液体整体柱（PIL-MCC/MNPs）。如图2所示，采用1-烯丙基-3-甲基咪唑双［（三氟甲基）磺酰基］酰亚胺（AM）为功能单体，ED为交联剂，将基于掺杂磁性纳米粒子的聚离子液体的毛细管整体柱作为固相微萃取管直接连接到六通阀，作为在线ME-IT-SPME系统的萃取介质。整体柱呈现相互连接的骨架和均匀的孔隙，与毛细管内壁紧密结合利于增强PIL-MCC/MNPs的稳定性。该萃取柱对目标分析物具有很高的亲和力和萃取能力。

**图2:**
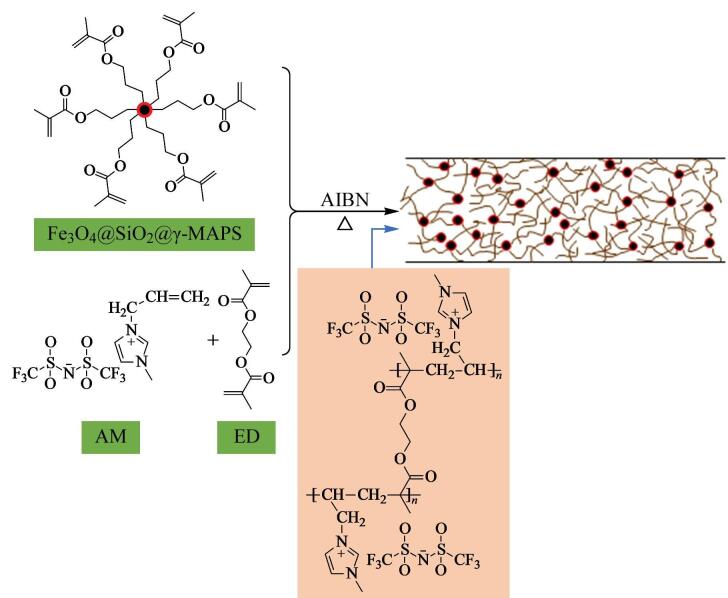
PIL-MCC/MNPs的制备原料和产物结构示意图^［15］^

Chen等^［16］^通过原位热引发聚合法在毛细管中制备了掺杂Fe_3_O_4_ NPs的整体柱（MCC@Fe_3_O_4_）。采用TEOS和*γ*-MAPS对Fe_3_O_4_进行改性，使其表面引入双键，从而能与整体柱紧密结合。以AM为功能单体、ED为交联剂，加入修饰后的磁性纳米颗粒连续超声使其分散均匀，聚合结束后，将其注入20 cm的熔融毛细管中，毛细管两端密封放入烘箱中进行热引发聚合。所制备的整体柱呈现菜花状形貌和多孔结构，比表面积为62.1 m^2^/g，利于分析物与萃取位点的充分接触；此外整体柱和毛细管内表面之间没有缝隙，Fe_3_O_4_ NPs均匀分散在整体柱中，磁化曲线表明其具有超顺磁性，保证了微萃取柱在外磁场下可以快速磁化和退磁。该微萃取柱在不明显损失萃取性能的情况下，可以重复使用30次以上，表现出良好的稳定性。

因此，采用原位热引发聚合法制备整体柱型固相微萃取管因具有良好的稳定性且制备简单而受到广泛应用。

Pang等^［17］^采用原位热引发聚合法制备了掺杂磁性纳米粒子的整体柱型固相微萃取管。首先合成改性的磁性纳米粒子Fe_3_O_4_@SiO_2_@*γ*-MAPS，再将1-乙烯基咪唑（VI）、ED混合超声后加入改性的磁性纳米粒子。将反应液导入预先用*γ*-MAPS修饰的熔融石英毛细管中，两端密封放入烘箱中聚合，成功合成了基于掺杂磁性Fe_3_O_4_纳米粒子的聚（乙烯基咪唑-乙二醇二甲基丙烯酸酯）的整体毛细管微萃取柱（VIED/MCMC）。Fe_3_O_4_ NPs分散在多孔结构的整体材料中并呈现均匀的球状，但在聚合过程中由于纳米粒子发生了部分沉积，导致该整体材料中出现少量纳米粒子堆积。整体柱与毛细管内壁紧密结合，孔径为300 nm，保证了微萃取柱良好的渗透性和稳定性。Pang等^［18］^也用4-乙烯基苯甲酸（VBA）为单体、ED为交联剂制备了掺杂磁性粒子的整体微萃取柱MBCC。合成的整体材料呈菜花状的多孔结构，孔径约为150 nm，保证了萃取过程的高渗透性。如图3所示，MNPs均匀地分散在萃取材料中，有利于在整体柱中产生微磁场，磁化曲线很好地证明了分散在萃取柱中的MNPs具有良好的磁化性能，有利于获得满意的萃取效果。该萃取柱可以在不明显损失萃取性能的情况下重复应用于富集目标分析物100次，具有良好的稳定性和可重复利用性。

**图3:**
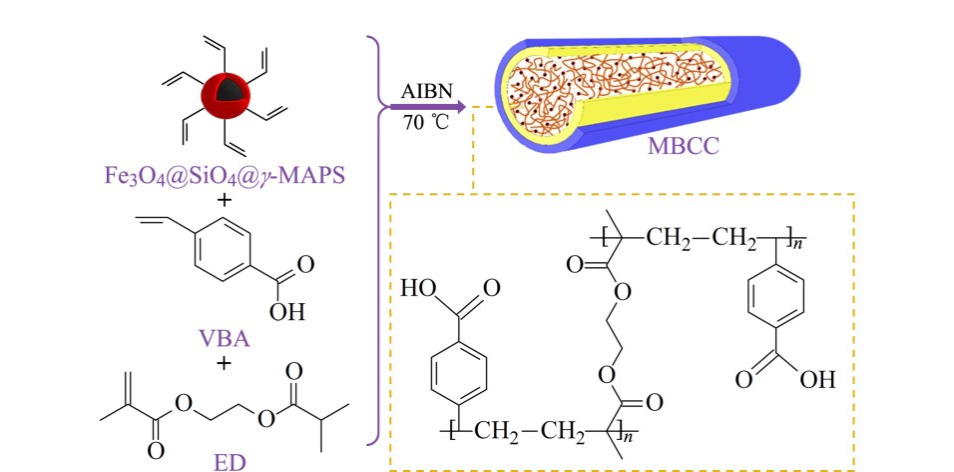
MBCC的聚合原理图^［18］^

Song等^［19］^制备了一种基于掺杂Fe_3_O_4_ NPs的整体柱型固相微萃取管（TSM@Fe_3_O_4_/CMC）。首先，采用*γ*-MAPS对Fe_3_O_4_纳米粒子进行预修饰引入双键使其与整体材料紧密结合。ED和二乙烯基苯（DB）为混合交联剂以改善其渗透性，以乙烯硼酸酐吡啶络合物和苯乙烯为双单体，加入预修饰的Fe_3_O_4_ NPs超声分散。在注射器的帮助下将聚合溶液注入内表面已被*γ*-MAPS修饰的熔融石英毛细管中，并用两个硅胶隔膜密封毛细管末端，再将其放于烘箱中70 ℃聚合12 h。整体材料呈现典型的多孔结构和交联球状，整体柱与毛细管内壁紧密连接，界面之间没有缝隙，大部分孔径为230 nm，比表面积为76.8 m^2^/g，表明有丰富的吸附位点。该萃取管可以连续在线富集分析物超过60个循环而没有明显的萃取性能损失，表明其具有良好的使用寿命和稳定性。

Song等^［20］^以1-烯丙基-3-甲基咪唑双三氟甲磺酰亚胺盐（AMI）和9-乙烯基蒽为双功能单体，DB为交联剂，在毛细管中原位制备了一种多孔聚合物掺杂磁性Fe_3_O_4_ NPs的整体微萃取柱，并将其作为ME-IT-SPME的固相微萃取管（见图4a）。该整体材料呈现颗粒状、交联结构和微米级孔隙，比表面积为70.3 m^2^/g，表明有丰富的吸附位点用于捕获分析物。所制备的微萃取柱具有高的渗透性，保证了ME-IT-SPME可以在较高的流速下萃取目标物，从而缩短分析时间。该萃取管在不明显损失萃取性能的情况下可重复使用100次，体现出了良好的制备重复性和使用寿命。Song等^［21］^还采用AM和3-丙烯酰胺基苯硼酸为功能双单体，DB和ED为混合交联剂，然后加入用*γ*-MAPS修饰的Fe_3_O_4_磁性纳米粒子。通过超声分散使其均匀分散在聚合溶液中，在注射器的辅助下将反应混合物引入到已用*γ*-MAPS预修饰的熔融石英毛细管中，将毛细管的两端密封进行原位聚合。整体柱与毛细管内表面紧密相连，界面间没有间隙。掺杂杂原子或官能团可以极大地改变吸附剂的表面性质，为获得更好的萃取效率提供有效的吸附位点。该微萃取柱含有丰富的官能团，碳、氮、硫、硼、铁等元素均匀地分布在整体柱中，比表面积约为62.1 m^2^/g。该萃取材料可以重复使用萃取富集分析物超过100个循环而富集性能没有明显损失，其优异的稳定性可以降低分析成本。Song等^［22］^还以含有咪唑基团的VI和AMI为功能单体，ED为交联剂，加入经*γ*-MAPS改性的磁性Fe_3_O_4_ NPs超声均质（见图4b）。将聚合溶液注入内表面经修饰的熔融毛细管中，并密封毛细管两端，制备了掺杂磁性纳米粒子的整体柱型固相微萃取管。该整体材料呈现交联球状和多孔结构，超大的孔径有利于传质。整体材料与毛细管内壁之间紧密连接，因为块体中可能存在较大的孔隙，所以比表面积相对较低（比表面积为7.13 m^2^/g）。该整体材料采用含有极性咪唑基团的VI作为功能单体，相比于9-乙烯基蒽作为单体，可以在反应中表现出更好的极性作用。咪唑分子中丰富的氮原子使其能形成强大的氢键，有利于萃取的进行。该整体材料可以连续用于在线富集所研究的络合物超过80个循环而没有明显的萃取性能损失。该整体材料良好的制备重复性和使用重复性利于对目标物的富集。

**图4:**
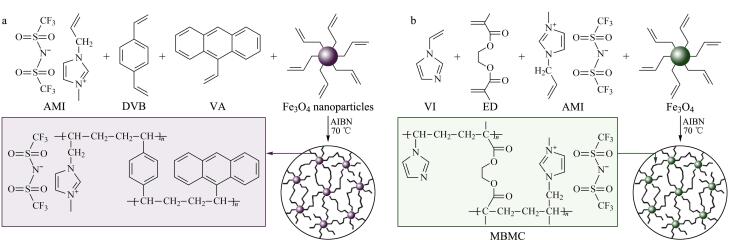
（a）TPM@MEC^［20］^和（b）MBMC^［22］^的原位合成图

综上所述，掺杂磁性纳米粒子聚离子液体的整体柱、二氧化硅包覆磁性颗粒的整体柱、磁性纳米粒子嵌入有机聚合物整体柱等新型吸附剂凭借使用寿命长和简便的合成步骤等独特优势已经广泛应用于ME-IT-SPME。优良的萃取材料应具有出色的化学稳定性和高的比表面积，如掺杂金属有机框架材料（MOFs）以增大比表面积从而提高萃取容量。开发更具潜力的吸附剂，如二氧化硅包覆磁铁核和层状双金属氢氧化物纳米片壳组成三组分磁性颗粒、采用免疫亲和材料功能化修饰多孔整体聚合物等，以提高萃取效率和萃取选择性。

## 2 磁增强管内固相微萃取技术的应用

磁增强管内固相微萃取技术是一种环保绿色和用途广泛的技术，可高效萃取和选择性富集多种类型的分析物，如农药、重金属离子、除草剂、防腐剂、药物分子等。与HPLC、HPLC-MS等分析技术联用可实现分析检测的自动化、缩短分析时间和提高检测灵敏度。

### 2.1 环境样品的分析检测

近年来，随着大气污染、土壤污染和河流污染等环境问题日益严峻，环境污染已经成为威胁人类社会发展的重要因素之一。大多数环境污染物如农药、除草剂、重金属离子、雌激素、防晒剂等具有致癌性和致畸性，因此对环境中污染物的检测和控制显得非常重要。由于环境样品的复杂性，样品前处理可以最大限度地减少干扰物引起的影响。考虑到许多污染物的沸点高、热稳定性差，不适合用气相色谱进行分析，磁增强管内固相微萃取与液相色谱在线联用是一个不错的选择。

#### 2.1.1 农药杀虫剂类

Moliner-Martinez等^［23］^利用二氧化硅负载的Fe_3_O_4_纳米颗粒作为萃取材料用于ME-IT-SPME，并与毛细管液相色谱（CLC）-二极管阵列检测结合用于分析痕量有机磷农药（OPs）。在本实验中，ME-IT-SPME应用于废水样品中OPs的富集，基于该萃取材料中十六烷基三甲基溴化铵胶束的烷基链与分析物的疏水作用和外加磁场力作用，分析物被吸附在材料中均匀的吸附位点，实验结果表明，毒虫畏和毒死蜱的平均回收率分别为94%±5%和97%±6%，LOD分别为50 ng/L和10 ng/L，萃取效率分别高达60%和84%，有力地证明了将外加磁场作用力引入IT-SPME能明显提高萃取效率和灵敏度。

磺酰脲类除草剂（SUHs）和三嗪类除草剂因其高效的除草能力在农业中被广泛使用，然而其降解产物在水体、土壤和生物体中的高毒性和持久性引起了人们的极大关注。为了高效萃取SUHs，Pang等^［17］^首次将ME-IT-SPME用于富集SUHs。在本实验中抗磁性的磺酰脲类除草剂在顺磁性介质中倾向于向磁场强度最小的方向移动，磁场的施加将萃取效率从36.8%～58.1%提高到82.6%～94.5%，有利于SUHs的富集。同时，将ME-IT-SPME和HPLC-DAD在线联用，对水体和土壤样品中SUHs进行了高效萃取和高灵敏度检测（如图5）。水、土壤样品中SUHs的LOD分别为0.030～0.15 μg/L和0.30～1.5 μg/kg，水样中SUHs的加标回收率为79.5%～114%，土壤样品中SUHs的加标回收率为80.6%～117%，并获得了满意的精密度。该方法比MSPE-HPLC-DAD^［24］^、SPME-HPLC/UV^［25］^等报道的方法有机溶剂消耗量更少，表明该在线联用方法在分析水样和土壤样品中的SUHs方面具有良好可行性。为了更加灵敏和低成本地检测环境水体中三嗪类除草剂的残留水平，Mei等^［26］^报道了ME-IT-SPME对环境水样中三嗪类除草剂的有效富集。在合成的掺杂磁性纳米颗粒的整体柱型固相微萃取管外缠绕磁线圈以便在吸附和洗脱步骤中利用外加电源施加可变磁场，考察了磁场强度、流速等因素对三嗪类除草剂吸附性能的影响。在最优条件下，对目标物的萃取效率为64.8%～99.7%，远高于IT-SPME（26.4%～84.9%）。将ME-IT-SPME与HPLC-DAD在线联用，对水样中的6种三嗪类除草剂进行检测（如图6），水样中6种三嗪类除草剂的LOD和LOQ分别为0.074～0.23 μg/L和0.24～0.68 μg/L，比SPE-HPLC/UV^［27］^等方法得到的LOD值更低，实现了萃取和色谱分析的自动化。该方法应用于农田、湖泊和河流等环境水样，获得了满意的加标回收率（70.7%～119%）。结果表明该研究可以成功地应用于检测环境水样中痕量三嗪类除草剂残留。

**图5:**
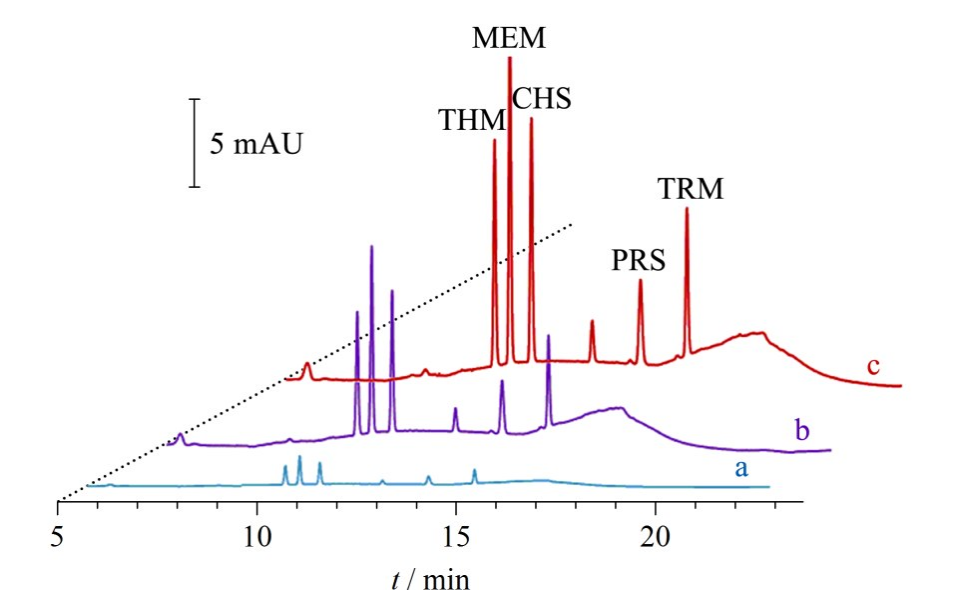
磺酰脲类除草剂（a）未富集和经（b）IT-SPME、（c）ME-IT-SPME富集后的色谱图^［17］^

**图6:**
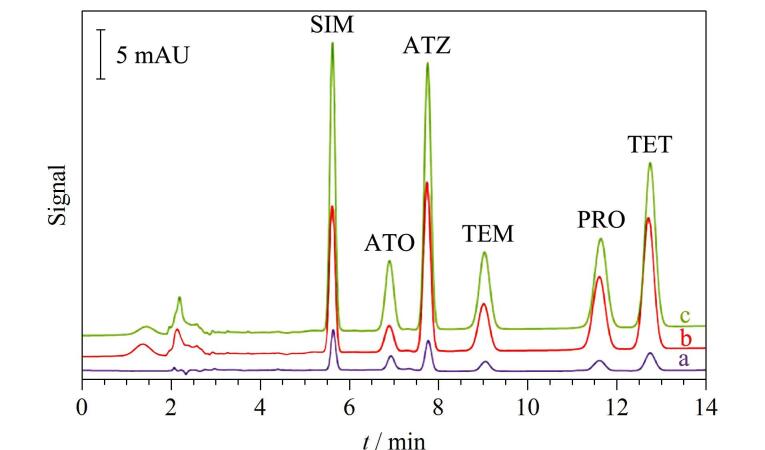
6种三嗪类除草剂在（a）富集前和经（b）IT-SPME、（c）ME-IT-SPME富集后的色谱图^［26］^

#### 2.1.2 重金属离子类

工业活动的加剧和废弃物的不正当处理使越来越多的重金属离子释放到环境中。重金属污染物的发生给人类和生物带来严重的健康威胁。Mei等^［14］^提出了用ME-IT-SPME有效富集重金属离子（HMIs）。选择Cu（Ⅱ）、Co（Ⅱ）和Hg（Ⅱ）3种重金属离子作为研究离子，与螯合剂二乙基二硫代氨基甲酸钠反应生成金属配位化合物以满足色谱分析的要求，并使金属离子具有紫外敏感性进而进行萃取。该研究选择含有硼酸基团的VA作为单体，从而利用吸附剂与金属配合物间的B*-*N配位作用萃取富集。采用HPLC-DAD在线测定并对影响萃取性能的关键参数进行了详细考察。基于疏水作用、*π*-*π*作用、B*-*N配位作用，在吸附和脱附过程中施加磁场使萃取效率从47%～65%提高到67%～89%，并缩短了分析时间。开发的ME-IT-SPME与HPLC-DAD在线联用方法应用于环境水和海产品中HMIs的测定，水样中HMIs的LOD值为0.004 3～0.035 μg/L，海产品样品中LOD值为0.69～4.9 μg/kg。该方法的LOD值比液液萃取-原子吸收光谱法（LLE-AAS）^［28］^、分散液液萃取（DLLE）-HPLC-UV^［29］^等报道的方法更低，加标回收率与LLE-AAS^［28］^、MSPE-电感耦合等离子体原子发射光谱法（ICP-AES）^［30］^等方法相当。ME-IT-SPME与HPLC-DAD在线联用，不仅节省了时间，而且提高了分析的准确性和精密度。因此，该方法不仅成功应用于检测环境水样中重金属离子的残留，而且为其他食品基质中重金属离子的检测也提供了良好的思路。

Pang等^［18］^将ME-IT-SPME与HPLC-DAD在线联用，提出了一种铬形态分析的新方法。为了满足色谱分离和紫外检测原理，Cr（Ⅲ）和Cr（Ⅵ）分别与吡咯烷二硫代氨基甲酸铵（APD）配位形成具有紫外吸收的Cr（Ⅲ）/APD和Cr（Ⅵ）/APD抗磁性有机金属配合物。APD中含有丰富的N、S原子，氢键、偶极-偶极作用和疏水作用可以对萃取作出贡献。在毛细管中原位制备了掺杂MNPs的整体柱型固相微萃取管，并在管外缠绕磁线圈以便在吸附和洗脱过程中施加不同强度和方向的磁场。研究详细考察了影响萃取性能的各种主要因素，实验结果表明配合物Cr（Ⅲ）/APD和Cr（Ⅵ）/APD的萃取效率分别为80.4%和86.2%（如图7）。在优化条件下，水样中配合物的检出限为0.002 0～0.005 9 μg/L，土壤中配合物的检出限为0.057～0.47 μg/kg。该在线联用方法比MSPE-火焰原子吸收光谱（FAAS）^［31］^、SPE-ICP/MS^［32］^等已报道的方法，有机溶剂消耗量更少，具有更高的灵敏度。环境水样和土壤样品中痕量Cr（Ⅲ）和Cr（Ⅵ）的成功定量检测证明了所提出的铬形态在线分析方法的可靠性和实用性。

**图7:**
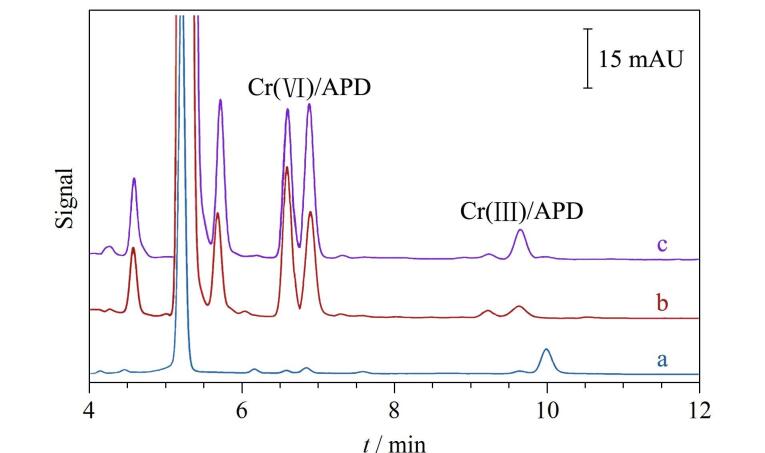
Cr（Ⅵ）/APD和Cr（Ⅲ）/APD在（a）富集前和经（b）IT-SPME、（c）ME-IT-SPME富集后的色谱图^［18］^

为了更加有选择性和高效地分析环境水体中痕量Hg形态，Song等^［19］^在毛细管中原位制备了基于掺杂Fe_3_O_4_ NPs的整体柱（TSM@Fe_3_O_4_/CMC），作为ME-IT-SPME的吸附剂，以富集和预浓缩与双硫腙配位形成螯合物的汞种。螯合物中含有丰富的N原子和苯环，吸附剂中含有丰富的苯环、B原子和吡啶基团，因此，吸附剂与螯合物之间的极性原子产生的偶极-偶极相互作用、芳香结构诱导的*π*-*π*堆积作用及B*-*N配位作用在富集过程中发挥了重要作用。螯合物表现出抗磁性，趋向于集中在磁场强度弱的区域，在萃取阶段施加磁场可以使汞螯合物的萃取效率从48.5%～75.3%提高到69.9%～94.4%。因此，在萃取阶段施加磁场有利于汞螯合物的富集。在优化的萃取参数下，将ME-IT-SPME与HPLC-DAD在线联用，实现了环境水中超痕量汞形态的准确分析。汞螯合物的LOD值为0.006 7～0.016 μg/L。该方法比DLLME-HPLC/DAD^［33］^、MSPE-HPLC-ICP/MS^［34］^等方法有机溶剂消耗量少，且样品用量更少。所提出的ME-IT-SPME与HPLC-DAD在线联用方法可以作为一种可靠有前途的策略来检测水和其他复杂样品中低含量汞的形态。

Song等^［35］^将ME-IT-SPME在线联用HPLC-DAD用于环境水体中Co（Ⅱ）和Ni（Ⅱ）的富集分析。Co（Ⅱ）和Ni（Ⅱ）与含有丰富配位原子和紫外敏感基团的螯合剂4-（2-吡啶偶氮）间苯二酚（PAR）反应生成抗磁性金属有机配合物，使金属离子具有抗磁性且满足色谱分离和紫外灵敏检测的要求。根据配合物的化学性质，选择富硼的VA和含有丰富咪唑基团的乙烯基-3-辛基咪唑四氟硼酸盐作为混合功能单体，制备了掺杂Fe_3_O_4_纳米粒子的整体柱（MCN）。在MCN的周围缠绕产生可变磁场的磁力线圈，用于Co-PAR和Ni-PAR螯合物的高效萃取（如图8）。通过优化进样流速、样品pH等萃取参数，达到了最佳条件。结果表明，基于磁场作用、极性相互作用、配位作用等，ME-IT-SPME对Co-PAR和Ni-PAR的萃取效率分别为91.7%和70.9%，高于IT-SPME（Co-PAR的萃取效率为52.2%，Ni-PAR的萃取效率为48.4%），充分证明在吸附和洗脱阶段施加磁场可以显著提高对Co-PAR和Ni-PAR的萃取效率。将建立的ME-IT-SPME-HPLC-DAD在线联用方法应用于河水、湖水、水库中Co（Ⅱ）和Ni（Ⅱ）的含量检测，Co（Ⅱ）和Ni（Ⅱ）的LOD为0.06～0.14 μg/L，加标回收率为81.4%～118%，RSD为2.0%～9.7%。与已报道的方法相比，该在线联用方法比SPME-HPLC/UV^［36］^、分散固相萃取（DSPE）-HPLC-DAD^［37］^等方法的样品用量和洗脱液用量更少，比SPME-ICP/MS^［38］^等方法分析时间更短，灵敏度更好。

**图8:**
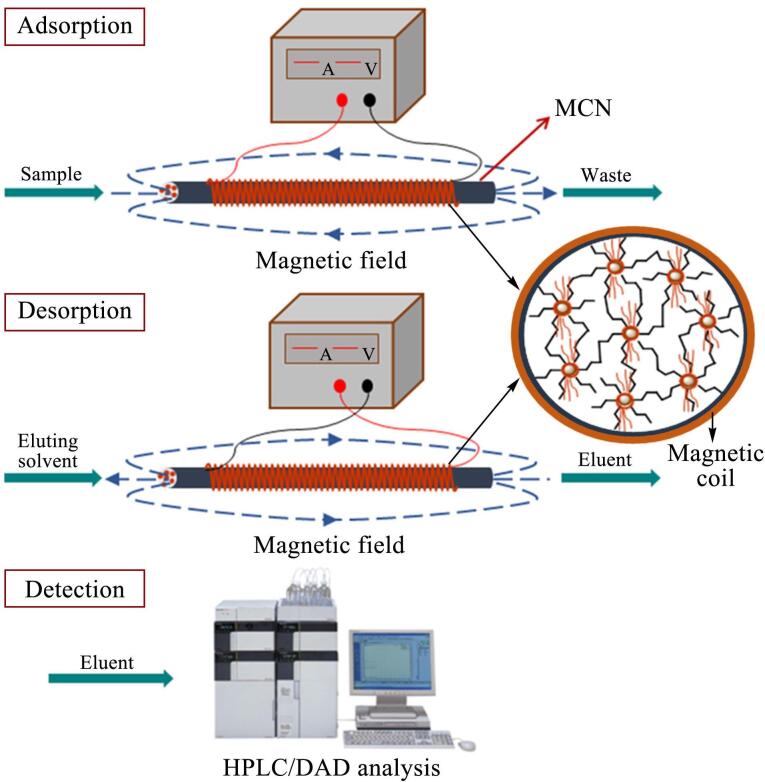
开发的ME-IT-SPME系统示意图^［35］^

#### 2.1.3 有机有毒污染物类

ME-IT-SPME也被用于富集分析环境中的有机有毒污染物。有机锡化合物（OTCs）作为一种难降解的有机有毒污染物，通过农业径流进入环境水体，对环境和人体健康构成威胁。基于实际需求，Song等^［20］^利用ME-IT-SPME与HPLC-DAD在线联用方法对水样和海产品中痕量OTCs进行分析。基于偶极-偶极作用、*π*-*π*作用、疏水作用，在吸附和洗脱阶段施加磁场，使OTCs的萃取效率从48.0%～77.0%提高到84.2%～99.7%，进一步提高了萃取效率和选择性（如图9）。在最优条件下，通过ME-IT-SPME联用HPLC，结合荧光检测（FD），建立了对OTCs的灵敏、自动定量方法。水样和海产品中OTCs的LOD值分别为0.003 0～1.5 μg/L和0.71～14 μg/kg，加标回收率为80.2%～116%，重复性良好（RSD小于10%）。该方法比DLLME-HPLC-MS/MS^［39］^、SPE-GC-ICP/MS^［40］^等方法样品用量少，比LLE-GC-MS^［41］^、SPE-HPLC-FD^［42］^等方法有机溶剂用量少，成本低。因此所建立的在线检测方法具有操作简单和生态友好等突出特点，可作为水体、海产品等复杂样品中痕量OTCs检测的良好选择。随着汽油消耗的不断增加，四乙基铅（TEL）作为一种具有剧毒性和致癌性的有机有毒污染物正通过各种途径释放到环境中，严重危害人类健康。Song等^［43］^将ME-IT-SPME在线联用HPLC-DAD的分离检测方法用于分析实际水样中的TEL；从图10可以看出，在吸附和洗脱过程中施加磁场有助于对TEL的选择性富集，与无磁场相比，萃取效率提高了52%。在优化条件下用所建立的ME-IT-SPME与HPLC-DAD在线联用方法检测自来水、湖水和水库中的痕量TEL，得到了满意的结果，水样中TEL的LOD为0.082 μg/L，RSD为6.3%～8.5%，线性范围为0.3～300 μg/L，加标回收率为80.6%～95.0%。ME-IT-SPME与HPLC-DAD在线联用方法为准确、可靠、自动化地检测水样品和其他复杂基质样品中痕量TEL提供了有力的技术支持。

**图9:**
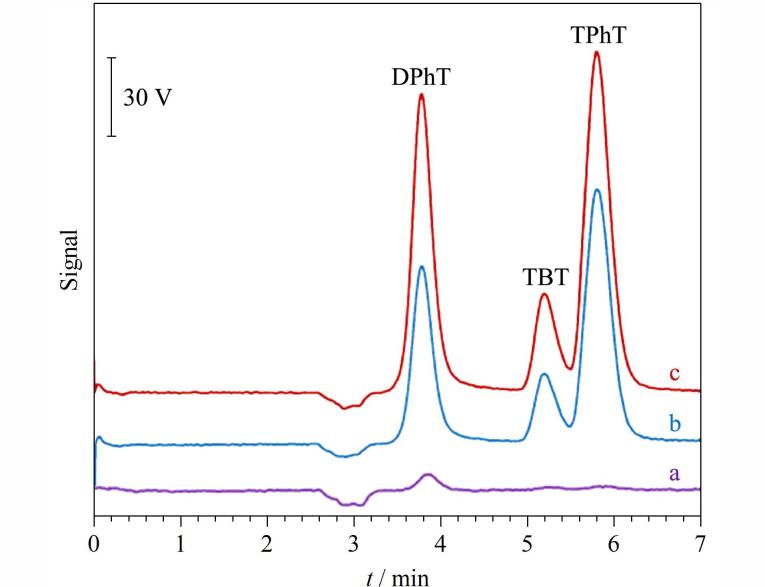
有机锡化物在（a）富集前和经（b）IT-SPME、（c）ME-IT-SPME富集后的色谱图^［20］^

**图10:**
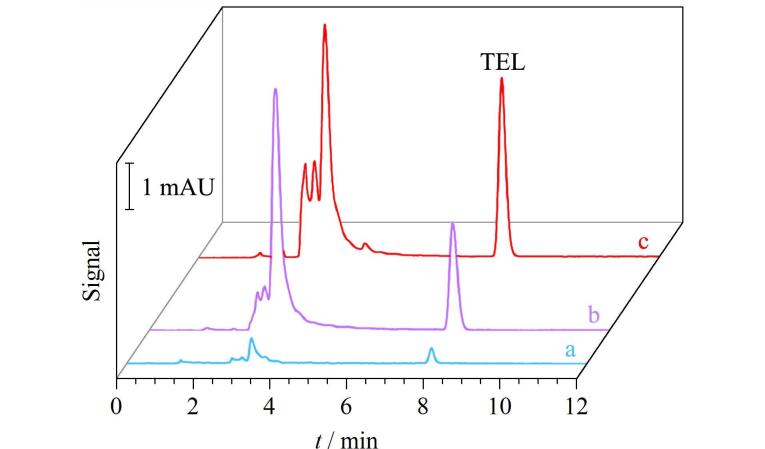
四乙基铅在（a）富集前和经（b）IT-SPME、（c）ME-IT-SPME富集后的色谱图^［43］^

#### 2.1.4 其他环境污染物

Mei等^［13］^利用ME-IT-SPME与HPLC-DAD在线联用方法分析水样中6种雌激素，所用整体毛细管柱（VPED-MCC/MNPs）被放置在允许施加可变磁场的磁线圈内部。研究考察了磁场强度、样品体积、吸附和洗脱流速等因素对ME-IT-SPME性能的影响。施加磁场后，目标分析物的萃取效率为70%～100%，明显高于IT-SPME得到的萃取效率（30%～65%），很好地证明了在萃取阶段施加磁场可以有效地提高萃取效率。将该在线联用方法应用于河水、井水和自来水中雌激素的检测，获得了较宽的线性范围（0.50～200 μg/L）、较低的检出限（0.06～0.25 μg/L）和满意的加标回收率（78.5%～121%）。

有机紫外防晒剂是一类具有强吸收紫外线作用的化妆品添加剂。但过量使用会刺激皮肤，引起过敏，且该类物质具有致畸性，通过生物链累积效应带来的巨大健康风险引起了人们的重视。为了更高效地富集分离水样中的有机紫外防晒剂，Mei等^［15］^开发了磁增强管内固相微萃取以有效富集有机紫外防晒剂，并对影响萃取效率的磁场强度、流速、有机溶剂体积和离子强度等因素进行全面优化。在优化条件下，基于磁场力作用、疏水相互作用、*π*-*π*作用、氢键、偶极-偶极相互作用，5种有机紫外防晒剂的萃取效率为44.0%～100%，远高于IT-SPME的萃取效率（11.3%～62.4%），LOD值为0.04～0.26 μg/L，LOQ为0.12～0.87 μg/L，线性范围为0.25～200.0 μg/L，RSD均小于10%。该方法比SPME-HPLC-UV^［44］^、单液滴微萃取（SDME）-HPLC-UV^［45］^等方法分析时间更短，LOD值比DLLME-GC-MS^［46］^等已报道的方法更低。所开发的ME-IT-SPME-HPLC-DAD在线联用方法成功应用于环境水体中痕量有机紫外防晒剂的检测，并获得了令人满意的结果。

Luo等^［21］^首次应用ME-IT-SPME富集水样中的无机硒（Se）。将合成在石英毛细管中掺杂磁性Fe_3_O_4_纳米颗粒的整体柱作为ME-IT-SPME的固相微萃取管，周围缠绕磁线圈以便在吸附和洗脱阶段施加磁场。为了满足磁微流控原理、色谱分离和紫外检测的要求，Se（Ⅳ）与邻苯二胺（OPA）配位反应生成抗磁性配位化合物。基于吸附剂与配合物间的偶极-偶极作用、B-N作用、*π*-*π*作用和氢键，在吸附过程中施加磁场有助于Se（Ⅳ）-OPA配合物的萃取，使萃取效率从83%提高到97%。在优化条件下，将ME-IT-SPME在线联用HPLC-DAD对环境水样中的Se（Ⅳ）和Se（Ⅵ）进行检测。在应用建立的方法之前，将Se（Ⅵ）预还原为Se（Ⅳ），定量总无机Se，并采用减法计算Se（Ⅵ）和Se（Ⅳ）的含量。Se（Ⅳ）的检出限为0.012 μg/L。通过对实际水样中Se（Ⅳ）和Se（Ⅵ）的测定，验证了该方法的可靠性，RSD小于9%，加标回收率为81.1%～116%。证明了该萃取柱对水样中Se具有高选择性识别能力。该在线联用方法比DLLE-电热原子吸收光谱（ETAAS）^［47］^、MSPE-ETAAS^［48］^等方法线性范围更宽，且样品和洗脱液用量低于大多数已报道的方法。因此，所提出的在线方法为富集检测痕量水平的Se（Ⅳ）和Se（Ⅵ）提供了一种经济且简便的途径。

综上所述，磁增强管内固相微萃取技术为富集检测环境样品中农药、除草剂、重金属离子、雌激素、有机有毒污染物、防晒剂等多种类型分析物提供了良好的思路，可以进一步改善萃取性能使其适用于更多分析物的高效富集。微型和快速准确是现代科学仪器的主要发展潮流。将ME-IT-SPME与便携式的先进分析仪器联用，使其应用于大气环境污染物的现场实时检测。在减少盐度干扰情况下，采用ME-IT-SPME与更精确的分析仪器联用对海水样品进行准确检测。

### 2.2 食品分析检测

在提倡绿色食品、健康食品的今天，还是存在着各种各样的食品安全隐患。在食品中添加防腐剂可以减缓食品在长期储存中发霉变质，延长食品的保质期，但防腐剂的过量使用会对人体造成慢性中毒伤害，且食品中微量元素的含量须在合理水平。这就要求科研工作者建立更为准确、简便、快速的食品样品富集检测方法。在食品分析检测领域，最大的问题在于食品样品中成分复杂，对待测组分干扰极大，目标分析物含量较低，这使ME-IT-SPME在线联用HPLC、LC-MS等分析方法成为解决有关食品分析检测问题的有力工具。

酚酸（PAs）是植物次生代谢的产物，因其被用作果汁中的防腐剂而受到特别关注。过度食用会引起荨麻疹、癌症。虽然固相微萃取^［49］^、磁性固相萃取^［50］^、搅拌棒吸附萃取^［51］^等技术对PAs的萃取性能良好，但是上述技术仍存在萃取介质稳定性差、萃取效率低等缺点。因此，引入更高效、准确的萃取方法对于分析复杂样品中的PAs十分必要。Chen等^［16］^开发了ME-IT-SPME用于果汁中酚酸类物质的富集分析。他们在毛细管中制备了嵌入Fe_3_O_4_ NPs的聚（1-烯丙基-3-甲基咪唑双三氟甲磺酰亚胺-乙二醇二甲基丙烯酸酯）整体柱（MCC@Fe_3_O_4_），将该微萃取柱用于对酚酸类物质的富集萃取，对影响萃取性能的各种参数进行了详细考察和优化，并用HPLC-DAD对洗脱液进行检测。结果表明，在吸附和洗脱阶段中施加磁场使分析物的萃取效率从44.9%～64.0%提高到78.6%～87.1%，疏水作用、氢键、*π*-*π*相互作用、偶极-偶极相互作用共同促进了PAs的富集，提高了萃取性能，获得了良好的灵敏度。在最佳萃取条件下，PAs的LOD为0.012～0.061 μg/L。该方法应用于果汁中5种分析物的同时定量检测，加标回收率为80.1%～116%，RSD为1.9%～9.8%，比SPME-GC-MS^［52］^等已报道的大多数方法样品用量更少，分析时间更短，节约了成本。因此，所开发的ME-IT-SPME是提高PAs在果汁和其他复杂样品中萃取效率的有效方法。Mei等^［53］^将基于整体柱的磁增强管内固相微萃取与HPLC-DAD在线联用，采用掺杂磁性纳米粒子的整体毛细管柱作为系统的固相微萃取管，应用于测定葡萄汁中对羟基苯甲酸酯类化合物。疏水作用、*π*-*π*相互作用和磁场力作用共同促进了吸附。在最佳萃取条件下，分析物的萃取效率为81.2%～88.2%。将该方法应用于葡萄汁样品和水样中对羟基苯甲酸酯类化合物的分析，葡萄汁样品中对羟基苯甲酸酯类化合物的LOD为0.019～0.051 μg/L，LOQ为0.062～0.17 μg/L，线性范围为0.10～200 μg/L，加标回收率为86.9%～108%。水样中对羟基苯甲酸酯类化合物的LOD为0.010～0.026 μg/L，线性范围为0.05～200 μg/L。该方法的LOD值比已报道的部分方法更低，分析时间更短^［54］^。结果表明，该方法简单、快速、灵敏、环保，适用于对羟基苯甲酸酯类化合物的痕量检测。

为了测定蔬菜等复杂样品中V的形态和含量，Song等^［22］^利用ME-IT-SPME与HPLC-DAD在线联用方法富集分析痕量V形态。与其他络合剂相比，络合剂乙二胺四乙酸（EDTA）不仅具有较高的螯合性能且含有紫外敏感基团，还可以在温和条件下与V快速配位。所以选择价格低廉的络合剂EDTA与V（Ⅳ）和V（Ⅴ）螯合形成具有紫外敏感性和抗磁性的有机络合物。在优化的条件下，将建立的ME-IT-SPME与HPLC-DAD在线联用方法对蔬菜和水样品中痕量V形态进行分析。水样和蔬菜样品中V（Ⅳ）-EDTA、V（Ⅴ）-EDTA的LOD分别为0.054～0.060 μg/L和1.4～1.5 μg/kg，加标回收率分别为82.5%～110%和83.8%～118%，线性范围分别为0.2～300 μg/L和5～5 000 μg/kg。该在线联用方法比DLLME-UV/Vis^［55］^、DLLME-ETAAS^［56］^等已报道的方法样品用量和有机溶剂用量更少，灵敏度更好。络合物中含有丰富的N、O原子，吸附剂中极性咪唑基团会与络合物中的O和N原子产生氢键和偶极-偶极相互作用，微萃取柱和目标物中的烷基基团也会产生疏水作用力，利于萃取。在吸附和洗脱阶段施加磁场，使V（Ⅳ）-EDTA和V（Ⅴ）-EDTA螯合物的萃取效率分别从55.7%～65.1%提高到80.7%～90.1%，萃取效率的明显提高说明了在吸附和洗脱阶段施加磁场可以提高对V物种的富集性能。因此，所提出的在线分析方法适用于蔬菜和水样品中痕量V形态的准确在线分析。

综上所述，磁增强管内固相微萃取技术对于富集检测食品样品中痕量目标物具有极大的潜力，避免了之前预处理过程中过滤、蒸发、再溶解衍生化等复杂操作步骤。面向食品安全检测是分析化学研究的一个重要方向，设计高性能、耐基质的萃取材料是ME-IT-SPME在食品分析中应用的重点。食品样品由于基质复杂，可通过更换有机单体、MOFs修饰等方式开发高性能的萃取材料，从而使具有理想选择性、优异萃取效能的功能化萃取材料应用于食品样品中杀虫剂、食品添加剂、农兽药残留等的富集，以提高分析结果的准确性。

### 2.3 生物医药分析检测

生物样品的研究对于分析工作者来说一直是个具有挑战性的任务，因为血液、尿液、唾液等样品基质复杂且分析物处于痕量或超痕量水平，所以不宜对此类样品直接进行分析。为了减少样品中其他物质的干扰并结合分析的实际要求，必须对样品进行分离、纯化、富集前处理。伴随着社会对动物源性食品需求量的增大，快速灵敏检测复杂生物样品中痕量药物残留成为分析领域的重点。

Moliner-Martínez等^［11］^合成了由SiO_2_负载Fe_3_O_4_纳米颗粒的毛细管柱，用于富集浓缩非甾体抗炎药物。高度多孔的网络涂层为萃取提供了有利的传质，目标药物为乙酰水杨酸、阿替洛尔、对乙酰氨基酚、双氯芬酸和布洛芬，这些有机分析物与吸附剂之间的主要相互作用机制归因于疏水相互作用和磁场力作用。在吸附和洗脱阶段施加不同方向的磁场，使得目标物的萃取效率为70%～100%，远高于IT-SPME的萃取效率（10%～30%）。进一步证明了在萃取阶段施加磁场力可以有效地提高萃取效率，特别是对极性化合物的萃取效率低。

畜牧业领域滥用抗生素导致药物在动物体内残留，人类食用残留有该类药物的动物食品会引起毒性作用和器官病变。喹诺酮类药物是一类人工合成的抗菌药物。氟喹诺酮类抗生素是喹诺酮类抗生素的衍生物。Manbohi等^［12］^利用基于十二烷基硫酸钠包被Fe_3_O_4_纳米颗粒填充管对尿样中的喹诺酮类药物进行ME-IT-SPME。由于静电排斥作用、疏水相互作用、磁场力作用，该萃取材料对喹诺酮药物有较强的萃取能力。将ME-IT-SPME与HPLC-UV在线联用对尿样中的喹诺酮类药物分析，并进一步研究显著影响因素。所有喹诺酮类药物的LOD为0.01～0.05 μg/L，呈现宽的线性范围（0.1～1 000 μg/L）。该在线联用方法比MSPE-CLC-UV^［57］^、SPE-LC-FLD/DAD^［58］^、中空纤维液相微萃取（HF-LPME）-HPLC-DAD^［59］^等已报道的方法萃取检测时间更短，线性范围更宽，LOD值更低。该研究不仅成功应用于检测尿样中喹诺酮类药物残留，而且为其他复杂基质样品中的喹诺酮类药物的残留检测提供了思路。

综上所述，磁增强管内固相微萃取技术现已成功地应用于生物样品中抗生素、药物残留等分析物的高效富集，获得了满意的结果。可以用限进分子印迹聚合物结合磁性纳米颗粒等方式进一步改善萃取材料的萃取性能，采用生物相容性萃取管用于血药浓度检测，单克隆抗体药物大分子有机结合磁性微球制备磁性探针并利用磁性分离释放出DNA链识别目标物检测，使其适用于更多类型分析物的高效富集分离，如中草药成分的富集分析、生物样品中农药残留、药物代谢动力学的研究，推动该技术应用于疾病诊断、临床治疗、生物分析化学等。

附表1（https://www.chrom-China.com）列举了磁增强管内固相微萃取技术在环境、食品和生物医药等领域的应用。

## 3 总结与展望

本文总结了自2012年ME-IT-SPME技术诞生以来，磁增强管内固相微萃取技术的材料制备及应用的研究进展。纳米材料、整体材料、磁性杂化材料等新型萃取材料已被作为ME-IT-SPME的吸附剂。利用吸附剂与分析物之间的多重相互作用高效萃取和选择性富集多种类型分析物，如有机农药、重金属离子、除草剂、雌激素、防晒剂、防腐剂、药物分子等。ME-IT-SPME联用色谱法灵敏分析目标物，不但提高了萃取效率和分析检测的准确性，而且减少了有机溶剂的消耗，缩短了分析时间，有效地节约了实验成本，已广泛应用于环境、食品、生物医药等领域。磁增强管内固相微萃取技术虽然有作为样品前处理技术的潜力，但仍处于研究阶段，存在有待改善的方面，比如高选择性的环保绿色萃取材料少、高磁场产生的热量造成局部升温不利于目标物的吸附、应用研究领域有限等。

基于本研究的结果，对于磁增强管内固相微萃取技术的进一步发展和改进，应该考虑以下几个重要的因素。第一，进一步研究磁性萃取材料的可重复利用性的机理，磁性萃取材料的可重复使用性和稳定性应该是关注的重点，并利用更精确的表征方法观察磁性萃取材料的微观结构，进而深入探究磁性萃取材料的形貌对萃取性能的影响。第二，研究开发一种具有免疫亲和性的萃取管，可用于高效选择性萃取生物大分子药物，如抗体、核酸。第三，通过磁性颗粒与限进分子印迹聚合物结合、层状双金属氢氧化物纳米外壳修饰二氧化硅包覆的磁铁核等开发新型理想萃取吸附剂，进一步提高萃取的性能。第四，以石墨烯嵌入多孔整体聚合物、MOFs掺杂到整体柱、IL为功能单体制备掺杂磁性纳米颗粒的整体柱等方式调节整体柱结构以增强整体柱的稳定性。第五，研发设计热干扰效应小的新型磁场调节装置，探究高磁场对萃取效率的影响。第六，将萃取装置与其他更灵敏的分析仪器在线联用以开发更准确的分析方法。第七，通过同时连接不同的萃取管，以实现同时富集多种分析物的目的。第八，将ME-IT-SPME与便携式的先进分析仪器联用实现对大气环境污染物的现场实时分析，有助于实时监测生物样品中信号变化。总之，磁增强管内固相微萃取技术展现了巨大的应用潜力。

## References

[1] Zheng J， Kuang Y X， Zhou S X， et al . Anal Chem， 2023， 95（1）： 218 36625125 10.1021/acs.analchem.2c03246

[2] Li X， Wen X X， Luo Z W， et al . Clin Chim Acta， 2023， 540： 117236 36716910 10.1016/j.cca.2023.117236

[3] Sun M， Feng J J， Han S， et al . Microchim Aata， 2021， 188（3）： 96 10.1007/s00604-021-04730-3 33619661

[4] Ali J， Tuzen M， Jatoi W B， et al . Food Chem， 2024， 437： 137908 37925781 10.1016/j.foodchem.2023.137908

[5] Costa Queiroz M E， Donizeti de Souza I， Marchioni C . TrAC-Trends Anal Chem， 2019， 111： 261

[6] Sun M， Bu Y N， Xin X B， et al . Microchem J， 2022， 181： 107699

[7] Ma R， Yu S S， Li Y F， et al . Front Environ Sci， 2024， 12： 1350170

[8] Sun M， Han S， Maloko Loussala H， et al . Microchem J， 2021， 166： 106263

[9] Winkleman A， Gudiksen K L， Ryan D， et al . Appl Phys Lett， 2004， 85（12）： 2411

[10] Watarai H， Namba M . J Chromatogr A， 2002， 961（1）： 3 12186388 10.1016/s0021-9673(02)00748-3

[11] Moliner-Martínez Y， Prima Garcia H， Ribera A， et al . Anal Chem， 2012， 84（16）： 7233 22861152 10.1021/ac301660k

[12] Manbohi A， Ahmadi S H . Anal Chim Acta， 2015， 885： 114 26231896 10.1016/j.aca.2015.05.030

[13] Mei M， Huang X J， Luo Q， et al . Anal Chem， 2016， 88（3）： 1900 26742590 10.1021/acs.analchem.5b04328

[14] Mei M， Pang J L， Huang X J， et al . Anal Chim Acta， 2019， 1090： 82 31655649 10.1016/j.aca.2019.09.028

[15] Mei M， Huang X J . J Chromatogr A， 2017， 1525： 1 29055526 10.1016/j.chroma.2017.09.065

[16] Chen H X， Song X C， Huang X J . J Sep Sci， 2021， 44（18）： 3418 34288429 10.1002/jssc.202100473

[17] Pang J L， Song X C， Huang X J， et al . J Chromatogr A， 2020， 1613： 460672 31727353 10.1016/j.chroma.2019.460672

[18] Pang J L， Chen H X， Huang X J . Microchem J， 2021， 164： 105956

[19] Song X C， Wu J Y， Pang J L， et al . J Hazard Mater， 2021， 411： 125141 33485231 10.1016/j.jhazmat.2021.125141

[20] Song X C， Luo Q， Huang X J . Anal Chim Acta， 2022， 1223： 340175 35998999 10.1016/j.aca.2022.340175

[21] Song X C， Luo S Y， Liu J， et al . The Analyst， 2022， 147（7）： 1499 35290422 10.1039/d1an02097h

[22] Song X C， Peng M M， Luo Q， et al . Talanta， 2023， 270： 125528 38118323 10.1016/j.talanta.2023.125528

[23] Moliner-Martinez Y， Vitta Y， Prima Garcia H， et al . Anal Bioanal Chem， 2014， 406（8）： 2211 24105458 10.1007/s00216-013-7379-y

[24] Ma J P， Xia Y， Lu W H， et al . J Chromatogr A， 2016， 1466： 12 27590086 10.1016/j.chroma.2016.08.065

[25] Yang J H， Zhou X M， Zhang Y P， et al . Adsorpt Sci Technol， 2017， 35（3）： 372

[26] Mei M， Huang X J， Yang X D， et al . Anal Chim Acta， 2016， 937： 69 27590547 10.1016/j.aca.2016.08.001

[27] Zhao R S， Yuan J P， Jiang T， et al . Talanta， 2008， 76（4）： 956 18656684 10.1016/j.talanta.2008.04.029

[28] Sorouraddin S M， Farajzadeh M A， Okhravi T . Talanta， 2017， 175： 359 28842003 10.1016/j.talanta.2017.07.065

[29] Werner J . Talanta， 2018， 182： 69 29501201 10.1016/j.talanta.2018.01.060

[30] Rofouei M K， Jamshidi S， Seidi S， et al . Microchim Acta， 2017， 184（9）： 3425

[31] Ghiasi A， Malekpour A . Microchem J， 2020， 154： 104530

[32] Hsu K C， Sun C C， Ling Y C， et al . J Anal At Spectrom， 2013， 28（8）： 1320

[33] Gao Z B， Ma X G . Anal Chim Acta， 2011， 702（1）： 50 21819859 10.1016/j.aca.2011.06.019

[34] He Y F， He M， Nan K， et al . J Chromatogr A， 2019， 1595： 19 30827698 10.1016/j.chroma.2019.02.050

[35] Song X C， Pang J L， Wu Y F， et al . Microchem J， 2020， 159： 105370

[36] Kaur V， Malik A K . Talanta， 2007， 73（3）： 425 19073051 10.1016/j.talanta.2007.04.003

[37] Farajzadeh M A， Yadeghari A . J Ind Eng Chem， 2018， 59： 377

[38] Rohanifar A， Rodriguez L B， Devasurendra A M， et al . Talanta， 2018， 188： 570 30029414 10.1016/j.talanta.2018.05.100

[39] Hu H， Tu W， Chen Y， et al . J Cancer， 2020， 11（8）： 2022 32127930 10.7150/jca.38981 PMC7052945

[40] González Toledo E， Benzi M， Compano R， et al . Anal Chim Acta， 2001， 443（2）： 183

[41] Quintas P Y， Alvarez M B， Arias A H， et al . Environ Sci Pollut Res， 2019， 26（8）： 7601 10.1007/s11356-019-04181-7 30659490

[42] González Toledo E， Compano R， Granados M， et al . J Chromatogr A， 2000， 878（1）： 69 10843546 10.1016/s0021-9673(00)00278-8

[43] Song X C， Meng X， Chen M S， et al . J Chromatogr A， 2023， 1700： 464040 37148567 10.1016/j.chroma.2023.464040

[44] Song W L， Guo M， Zhang Y D， et al . J Chromatogr A， 2015， 1384： 28 25662065 10.1016/j.chroma.2015.01.059

[45] Vidal L， Chisvert A， Canals A， et al . Talanta， 2010， 81（1）： 549 20188961 10.1016/j.talanta.2009.12.042

[46] Clavijo S， Avivar J， Suárez R， et al . J Chromatogr A， 2016， 1443： 26 27016119 10.1016/j.chroma.2016.03.036

[47] Uluozlü D . At Spectrosc， 2019， 40（4）： 133

[48] Kalantari H， Manoochehri M . Microchim Acta， 2018， 185（3）： 196 10.1007/s00604-018-2731-8 29594729

[49] Peng L Q， Yi L， Yang Q C， et al . Sci Rep， 2017， 7（1）： 7496 28790408 10.1038/s41598-017-07840-2 PMC5548748

[50] Shi S， Fan D， Xiang H， et al . Food Chem， 2017， 237： 198 28763986 10.1016/j.foodchem.2017.05.086

[51] Xu J， Chen B， He M， et al . J Chromatogr A， 2013， 1278： 8 23336943 10.1016/j.chroma.2012.12.061

[52] Tashakkori P， Tagac A A， Merdivan M . J Chromatogr A， 2021， 1635： 461741 33253998 10.1016/j.chroma.2020.461741

[53] Mei M， Pang J L， Huang X J . Anal Methods， 2018， 10（17）： 1977

[54] Chen W， Huang H F， Chen C E， et al . Chemosphere， 2016， 163： 99 27522181 10.1016/j.chemosphere.2016.07.080

[55] Uslu M， Ulutürk H， Yartasi A， et al . Toxicol Environ Chem， 2013， 95（10）： 1638

[56] Dadfarnia S， Haji Shabani A M， Dehghanpoor F . J Sep Sci， 2016， 39（8）： 1509 26891590 10.1002/jssc.201501301

[57] Xu S， Jiang C， Lin Y X， et al . Microchim Acta， 2012， 179（3）： 257

[58] Marazuela M D， Moreno Bondi M C . J Chromatogr A， 2004， 1034（1）： 25 15116911 10.1016/j.chroma.2004.02.022

[59] Ramos Payán M， Bello López M A， Fernández Torres R， et al . J Pharm Biomed Anal， 2011， 55（2）： 332 21353435 10.1016/j.jpba.2011.01.037

